# Tyrosine Hydroxylase Binding to Phospholipid Membranes Prompts Its Amyloid Aggregation and Compromises Bilayer Integrity

**DOI:** 10.1038/srep39488

**Published:** 2016-12-22

**Authors:** Anne Baumann, Ana Jorge-Finnigan, Kunwar Jung-KC, Alexander Sauter, Istvan Horvath, Ludmilla A. Morozova-Roche, Aurora Martinez

**Affiliations:** 1 Department of Biomedicine, University of Bergen, 5009 Bergen, Norway; 2 Division of Psychiatry, Haukeland University Hospital, 5021 Bergen, Norway; 3 K.G. Jebsen Centre for Neuropsychiatric Disorders, University of Bergen, 5009 Bergen, Norway; 4 Department of Clinical Dentistry, University of Bergen, 5009 Bergen, Norway; 5 Department of Medical Biochemistry and Biophysics, Umeå University, 90187 Umeå, Sweden

## Abstract

Tyrosine hydroxylase (TH), a rate-limiting enzyme in the synthesis of catecholamine neurotransmitters and hormones, binds to negatively charged phospholipid membranes. Binding to both large and giant unilamellar vesicles causes membrane permeabilization, as observed by efflux and influx of fluorescence dyes. Whereas the initial protein-membrane interaction involves the N-terminal tail that constitutes an extension of the regulatory ACT-domain, prolonged membrane binding induces misfolding and self-oligomerization of TH over time as shown by circular dichroism and Thioflavin T fluorescence. The gradual amyloid-like aggregation likely occurs through cross-β interactions involving aggregation-prone motives in the catalytic domains, consistent with the formation of chain and ring-like protofilaments observed by atomic force microscopy in monolayer-bound TH. PC12 cells treated with the neurotoxin 6-hydroxydopamine displayed increased TH levels in the mitochondrial fraction, while incubation of isolated mitochondria with TH led to a decrease in the mitochondrial membrane potential. Furthermore, cell-substrate impedance and viability assays showed that supplementing the culture media with TH compromises cell viability over time. Our results revealed that the disruptive effect of TH on cell membranes may be a cytotoxic and pathogenic factor if the regulation and intracellular stability of TH is compromised.

Biological membranes are mainly composed of phospholipids and associated proteins, as well as polysaccharides, where the phospholipids organize in a bilayer to which the proteins associate, retaining their mobility and flexibility, and performing their function as transporters, enzymes, signal transducers or molecular scaffolds^1^,^2^. Integral proteins are firmly bound across the lipid membrane, whilst peripheral proteins reversibly interact with the surface via several specific mechanisms such as lipid and helical anchors, partial unfolding, phosphorylation, and stearoyl-arachidonoyl segregation^3^,^4^. Under normal cellular conditions, these interactions contribute to protein re-location and regulation of their functional cycle without perturbing membrane integrity or cell viability.

In addition to delimiting cell and subcellular structures, biological membranes modulate a wide range of physiological and pathological processes and, therefore, the loss of cell membrane integrity can be harmful, affecting normal cell functioning and survival. Dysregulated interactions between proteins and membranes can result in disruptive perturbations of lipid membrane potentially accompanied with protein unfolding and/or aggregation^5^,^6^. For instance, amyloid aggregates of α-synuclein (α-syn), a protein widely distributed in neuronal tissue and associated with neurological Parkinson’s (PD) and Alzheimer’s (AD) diseases, lead to membrane disruption^7^,^8^. The physiological function of α-syn is not yet clear, but it plays a role in the stabilization of synaptic vesicles^9^ and is involved in the interactions with synthetic lipid bilayers, membranous cell fractions and organelles, including synaptic vesicles^10^. The pathological role of α-syn is associated with its amyloid aggregation and fibrillation upon overexpression or mutation, with amyloid oligomers being most effective disruptors of membranes^5^. Other proteins and peptides can aggregate and affect the membrane permeability and, consequently, stimulate the membrane channel activity and disrupt the cellular ionic homeostasis *in vivo*^11^,^12^. Therefore, understanding the molecular basis of protein misfolding and aggregation in the context of membrane-protein interactions will help to elucidate the pathomechanisms in neurological diseases such as AD or PD.

One of the hallmarks of PD is the decrease of tyrosine hydroxylase (TH) levels and the loss of dopaminergic neurons that express this enzyme in the substantia nigra pars compacta^13^,^14^, leading to a reduction of dopamine levels in the striatum. TH is found in the central and peripheral sympathetic nervous systems as well as in the adrenal medulla, where it catalyzes the hydroxylation of L-tyrosine to L-dihydroxyphenylalanine (L-Dopa)^15^. The reaction is dependent on the cofactor tetrahydrobiopterin (BH_4_), molecular oxygen and ferrous iron. This initial reaction is the rate-limiting step in the production of catecholamines, dopamine, norepinephrine and epinephrine, which function as neurotransmitters and hormones. TH is a homotetramer where each subunit contains three domains – the N-terminal regulatory ACT domain, the central catalytic domain and the C-terminal tetramerization domain^16^,^17^. TH is a cytoplasmic protein but it was also found to be membrane-bound at the brain nerve endings and synaptic vesicles^18^,^19^. There is scant knowledge on the structural determinants and conformational changes occurring upon TH membrane binding, though it has been shown that the direct interaction with negatively charged membranes depends on N-terminal extension to the ACT domain^20^,^21^.

Mutations in TH are associated with neurological disorder of TH deficiency (THD)^22^, with phenotype ranging from mild L-DOPA responsive dystonia (type A)^23^ to a progressive encephalopathy with L-DOPA-unresponsive parkinsonism (type B)^24^. Furthermore, it has also been suggested that TH plays a pathogenic role in PD, either through generation of reactive oxygen species (ROS) during catalysis, especially by uncoupled reactions, and/or as a consequence of enzyme dysregulation^25^,^26^,^27^. However, an involvement of wild-type TH in the pathogenesis of neurological disorders, and notably PD, has not yet been proven.

Based on the propensity of TH to bind to negatively charged membranes^20^,^21^ in this work we have studied the effects that this interaction may have on membrane integrity, enzyme conformation and cellular viability. We have examined the interaction of isoform 1 of human TH (hTH1) with negatively charged liposomes using large unilamellar vesicles (LUVs). The effects of membrane binding on hTH1 conformation and oligomeric state were investigated by circular dichroism (CD), dynamic light scattering (DLS) and fluorescence spectroscopy. Atomic force microscopy (AFM) was applied to monitor distortions in lipid monolayer consistent with hTH1 aggregation. Some insights into the biological consequences of hTH1 interaction with membranes were gained in experiments with PC12 cells treated with neurotoxin 6-hydroxydopamine (6-OHDA), as well as by measuring the effect of hTH1 on mitochondrial membrane potential. The results on cell viability assays with HEK293 cells demonstrated that hTH1 binding compromised cell viability over time, probably through apoptotic or necroptotic pathways, triggered by the disruptive binding of enzyme to membranes. TH is abundant in dopaminergic cells of the *substantia nigra*, and our results provide novel insights into that may contribute to understand pathogenic mechanisms leading to marked degeneration in PD via TH-associated pathways.

## Results

### Membrane Permeabilization Caused by hTH1

**Figure 1:**
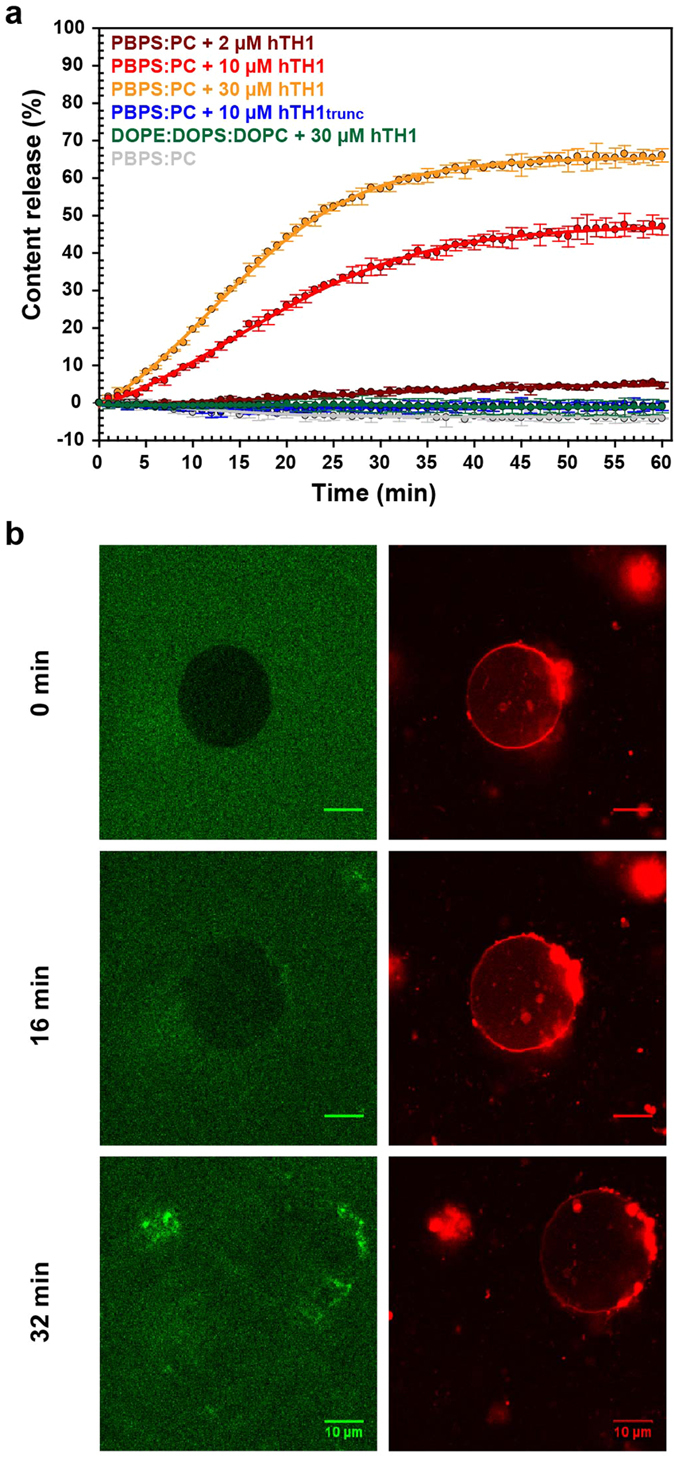
Effect of hTH1 binding on membrane integrity. (**a)** Disruption of membrane integrity monitored by leakage of fluorophore (ANTS). Vesicle leakage over time at 37 °C in 10 mM Na-Hepes, 150 mM NaCl, pH 7.0 induced by hTH1 at 2 μM (dark red), 10 μM (red) and 30 μM (orange) subunit or 10 μM hTH1_trunc_ (blue) in the presence of PBPS:PC liposomes (0.8 mM phospholipid, pH 7.0). DOPE:DOPS:DOPC liposomes showed no leakage after adding 30 μM hTH1 (green). Buffer controls in the absence of protein (grey) showed no effect on the PBPS:PC membrane integrity over time (same for DOPE:DOPS:DOPC, data not shown). Averaged values (±SD) of three parallel measurements are presented. Curve fitting for hTH1 and hTH1_trunc_ was done by nonlinear regression with equation for a sigmoidal function using 3 or 4 parameters, which provided maximum leakage values (±SE) of 5.8 ± 0.5%, 59.2 ± 1.5% and 80.7 ± 1.3% for 2 μM, 10 μM and 30 μM hTH1, respectively. (**b)** Confocal microscopy images monitoring the permeation of single unilamellar vesicles. Rhodamine B labelled PBPS:PC GUVs (right column) were embedded in calcein enriched (left column) agarose gel in 10 mM Na-Hepes, 150 mM NaCl, pH 6.0. Membrane permeability was monitored for 32 min after 20 μM hTH1 was added to the chamber. Images at different time points are presented. At 16 min, most of the calcein has entered the GUV while the morphology of the membrane was retained.

Previous studies have determined the interaction of TH and its N-terminal sequence with negatively charged membranes^20^,^21^,^28^, however the effect of this interaction on membrane integrity has not been investigated. We assessed the effect of TH on the integrity of negatively charged membranes, using purified recombinant hTH1, which is the most abundant splicing isoform of TH in brain and adrenal medulla^17^, and measuring the fluorescence of released 8-aminonaphtalene-1,3,6-trisulphonic acid, disodium salt (ANTS) from LUVs, which will also be referred here to as liposomes. This fluorophore is quenched by p-xylene-bis-N-pyrimidium bromide (DPX) while inside the liposome, but turns fluorescent upon release^29^. This assay was performed using large unilamellar vesicles made of PBPS:PC which represents the general phospholipid composition of neuronal membranes^20^,^30^ and concentrations of hTH1 up to 30 μM subunit. A hTH1-concentration dependent release of fluorophore was observed over time after hTH1 was added to solutions of the liposomes (Fig. 1a). Truncated hTH1 (hTH1_trunc_) prepared by limited proteolysis and lacking the N-terminal extension to the regulatory ACT domain (~5 kDa per monomer)^24^ induced no leakage after 1 h compared to the effect produced by full-length hTH1 (Fig. 1a). We also tested the effect of hTH1 on the integrity of liposomes made of DOPE:DOPS:DOPC (5:3:2), which have been argued to better approach the composition of synaptic vesicle membranes^31^,^32^. No leakage could be observed from DOPE:DOPS:DOPC liposomes, although we corroborated by DLS that hTH1 actually bound to these negative charged liposomes (data not shown).

The membrane-disruptive activity of hTH1 was also studied by confocal microscopy using GUVs partially immobilized in 0.5% agarose, in which we could visualize if TH interaction affected the lamellarity and caused changes and morphology and the circularity of the vesicles. We selected the PBPS:PC composition, which was readily prone to permeability disruption, as seen from the leakage experiments in LUVs. Calcein was added to the samples to stain the extravesicular space, followed by hTH1. Initially, no calcein was observed inside GUVs, indicating that the membranes were intact. After ca. 8 min since hTH1 was added, calcein slowly began to be visible inside GUVs. After 16 min, and notably at 32 min, the signal from calcein was almost the same in the interior as in the exterior of GUVs, despite the fact that GUVs did not collapse (Fig. 1b). Nevertheless, the membrane became wrinkled and small internal membrane structures were developed. Controls without hTH1 showed that GUVs were stable over time and calcein stayed in their exterior (data not shown).

### hTH1 Conformation upon Membrane Interaction

**Figure 2:**
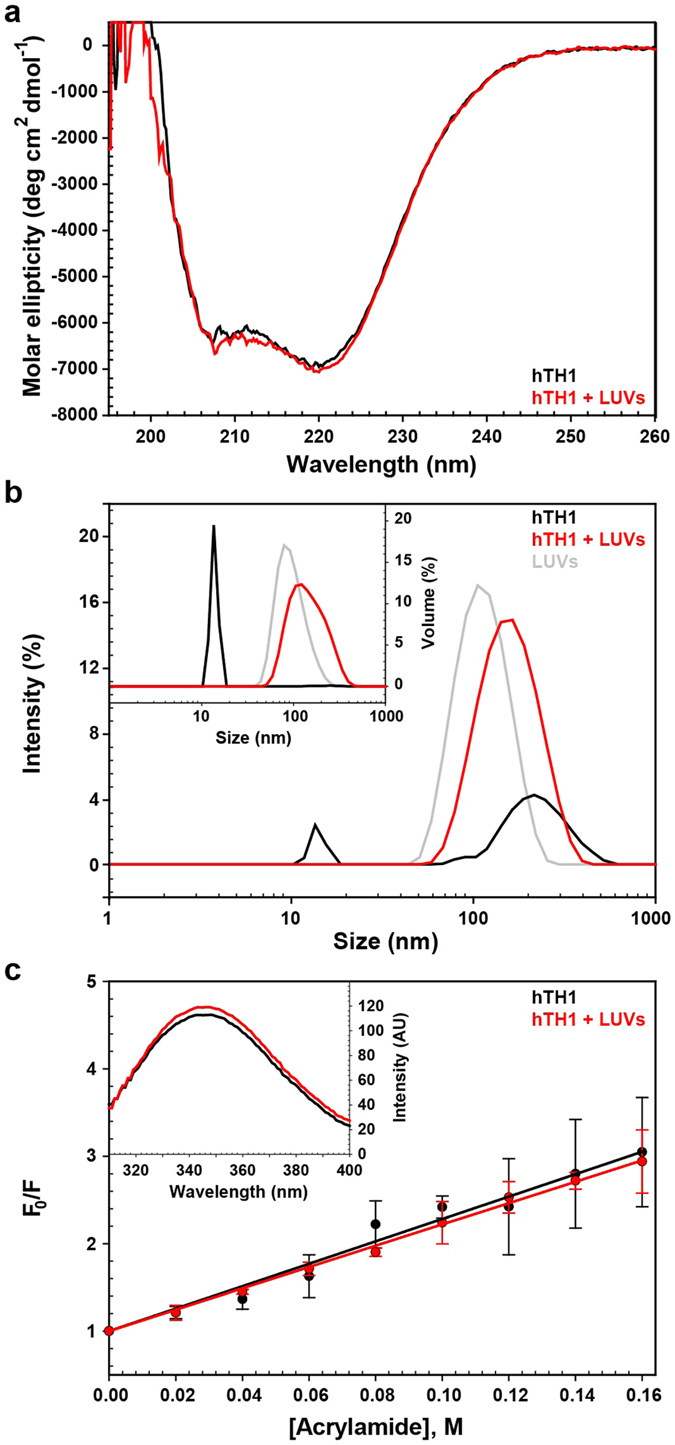
hTH1 conformation upon PBPS:PC liposome interaction. (**a)** Far-UV CD spectra of hTH1 in 10 mM Na-Hepes, 150 mM KF, pH 7.0 (4 μM subunit), without liposomes (black) and with liposomes (0.32 mM phospholipid; red) at 37 °C. (**b)** DLS intensity particle size distribution of hTH1 (4 μM subunit; black) at 37 °C in 10 mM Na-Hepes, 150 mM NaCl, pH 7.0. Liposomes (0.32 mM phospholipid) without (grey) and with hTH1 (4 μM subunit; red) at 37 °C. Inset: Volume size distribution of hTH1 (black), liposomes (grey) and both together (red) at 37 °C. (**c)** Stern-Volmer plots for quenching of tryptophan fluorescence of hTH1 by acrylamide in 10 mM Na-Hepes, 150 mM NaCl, pH 7.0 (black) and in the presence of liposomes (red) at 37 °C. The final concentration of hTH1 and phospholipid liposomes were 2 μM (subunit) and 0.16 mM, respectively. Inset: Tryptophan fluorescence intensity of hTH1 (2 μM subunit) was measured alone (black) and in the presence of liposomes (0.16 mM phospholipid; red). hTH1 free-blanks were measured in parallel and curves are blank-subtracted. The curves present an average of three independent measurements each containing three parallels. Experiments (**a–c**) were started immediately after preparation of the samples.

In order to investigate the possible conformational effects on hTH1 upon membrane interaction we monitored the secondary structure of hTH1 in the presence and absence of PBPS:PC liposomes. As seen by CD at the selected pH (7.0), temperature (37 °C) and protein:lipid ratios that provide total binding of protein to membrane^21^ (Fig. 2a), good signal-to-noise ratio was obtained. The far-UV CD spectrum of hTH1 was characteristic of a well-folded helical structure with a double minimum at 222 nm and 208 nm, and did not change significantly in the presence of the LUVs at 37 °C, when CD spectra were recorded immediately after mixing (Fig. 2a). The propensity of hTH1 to undergo self-oligomerization upon membrane interaction was then investigated by DLS. As observed by intensity distribution measurements, the purified hTH1 samples consist of two populations with an apparent hydrodynamic diameter of 13.9 ± 0.5 nm and 239.9 ± 54.2 nm (Fig. 2b). The size of the smaller protein population corresponds well to the dimensions of tetrameric enzyme, as assessed in structural models of full-length TH (∼12.4 nm)^16^, and verified by small-angle X-ray scattering^33^, whereas the population with larger diameter corresponds to highly scattering aggregates. Conversion into the volume size distribution corrects for size-dependent scattering effect and only reveals the tetrameric population (Fig. 2b, inset), indicating that these aggregates are present in minor amounts. The LUVs had a size of 97.7 ± 3.0 nm and 116.3 ± 2.8 nm (volume and intensity peak size, respectively). Interestingly, in the sample of hTH1 together with liposomes taken at 37 °C, we observed no peak corresponding to tetrameric hTH1, but a broad peak with a slightly larger size than liposomes alone, suggesting that hTH1 readily associates with liposomes.

In order to investigate possible conformational changes of hTH1 and some indication of enzyme positioning in the bilayer upon the initial stage of membrane binding, the intrinsic emission tryptophan fluorescence of hTH1 and its quenching by acrylamide were investigated. The fluorescence of hTH1 (λ_em max_ and intensity) is not affected by liposome binding (Fig. 2c, inset; λ_em max_ = 344.7 ± 0.1 nm for hTH1 and 344.9 ± 0.2 nm for hTH1 in the presence of liposomes). hTH1 contains three tryptophans in each subunit in the tetramer, W165 is located in the boundary between the catalytic and the regulatory domain and W232 and W371 are positioned in the catalytic domain. Stern-Volmer plots showed a similar decrease in tryptophan fluorescence with increasing acrylamide concentration both in the absence or presence of liposomes (Fig. 2c), with *K*_sv_ values of 12.8 ± 3.5 M^−1^ for hTH1 and 12.2 ± 1.4 M^−1^ for hTH1 in the presence of liposomes. Thus, the tryptophans appear accessible to the quencher to the same degree in membrane-bound hTH1 as in free hTH1, indicating that this binding is superficial, rather than reflecting deep insertion into the bilayer membrane.

### hTH1 Amyloid Aggregation Induced by Prolonged Interaction with Membrane

**Figure 3:**
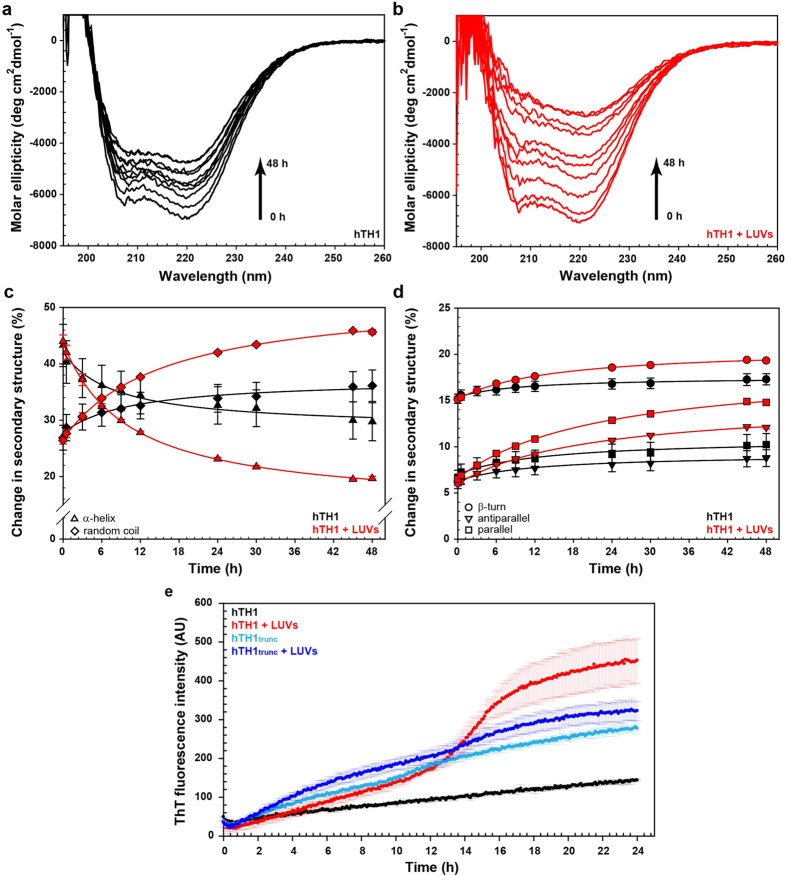
Structural changes of hTH1 upon PBPS:PC liposome interaction over time. (**a,b)** Far-UV CD spectra of hTH1 in 10 mM Na-Hepes, 150 mM KF, pH 7.0 (4 μM subunit), without liposomes (**a**) and with liposomes (0.32 mM phospholipid; (**b**)) incubated over 48 h at 37 °C. (**c,d)** Estimated secondary structure by CDNN. Changes of random coil and α-helical structure (**c**); and β-turn and β-sheets (**d**) of hTH1 (black) and hTH1 in the presence of liposomes (red). (**e)** Time dependent ThT fluorescence intensity monitored at 482 nm, with excitation at 440 nm, at 37 °C. Representative profiles for 10 μM hTH1_trunc_ in the absence (light blue) and presence (blue) of PBPS:PC liposomes (0.8 mM phospholipid), or with 10 μM full-length hTH1 in the absence (black) and presence of liposomes (red). Controls with liposomes without enzymes were performed under identical conditions and subtracted. Averaged curves ( ± SD) of three parallel measurements are presented.

Our measurements with CD and DLS during initial interaction of hTH1 with liposomes (Fig. 2a) indicated that the loss of membrane permeability was not accompanied by large conformational changes or substantial aggregation of hTH1. We investigated the time-dependent enzyme conformational change, oligomerization and/or aggregation upon membrane interaction by CD (Fig. 3). Far-UV CD spectra of hTH1 and hTH1 in the presence of PBPS:PC liposomes showed a decrease of α-helix content over time and the effect became more pronounced when the enzyme was bound to the membrane (Fig. 3a,b). As estimated by the CDNN algorithm^34^ hTH1 underwent a larger decrease of α-helical structure when bound to liposomes for 48 h compared to hTH1 alone (Fig. 3c). On the other hand, the β-turn and β-sheet contents (both parallel and antiparallel) increased over time, with larger increase estimated for hTH1 in the presence of liposomes than when alone (Fig. 3d).

In order to investigate if the CD-monitored conformational change involved a cross-β type of aggregation, we monitored the time dependent increase in Thioflavin T (ThT) fluorescence, which is associated with formation of inter-chain β-sheets^35^,^36^. As seen in Fig. 3e, hTH1 alone induces a very slow increase of ThT fluorescence, whereas the presence of PBPS:PC liposomes accelerates the fluorescence increase, with a notable lag-phase and an accelerated rate at 12 h at the selected conditions. This supports the implication of cross-β structures upon membrane binding of hTH1. Interestingly, the initial rise of ThT fluorescence intensity was also observed with hTH1_trunc_ alone (Fig. 3e). However, the rate is not much affected by the presence of liposomes, indicating the importance of the N-terminal tail of hTH1 in binding and aggregation/fibrillation on membrane.

### hTH1 Amyloid Self-Assembly Monitored by Atomic Force Microscopy

**Figure 4:**
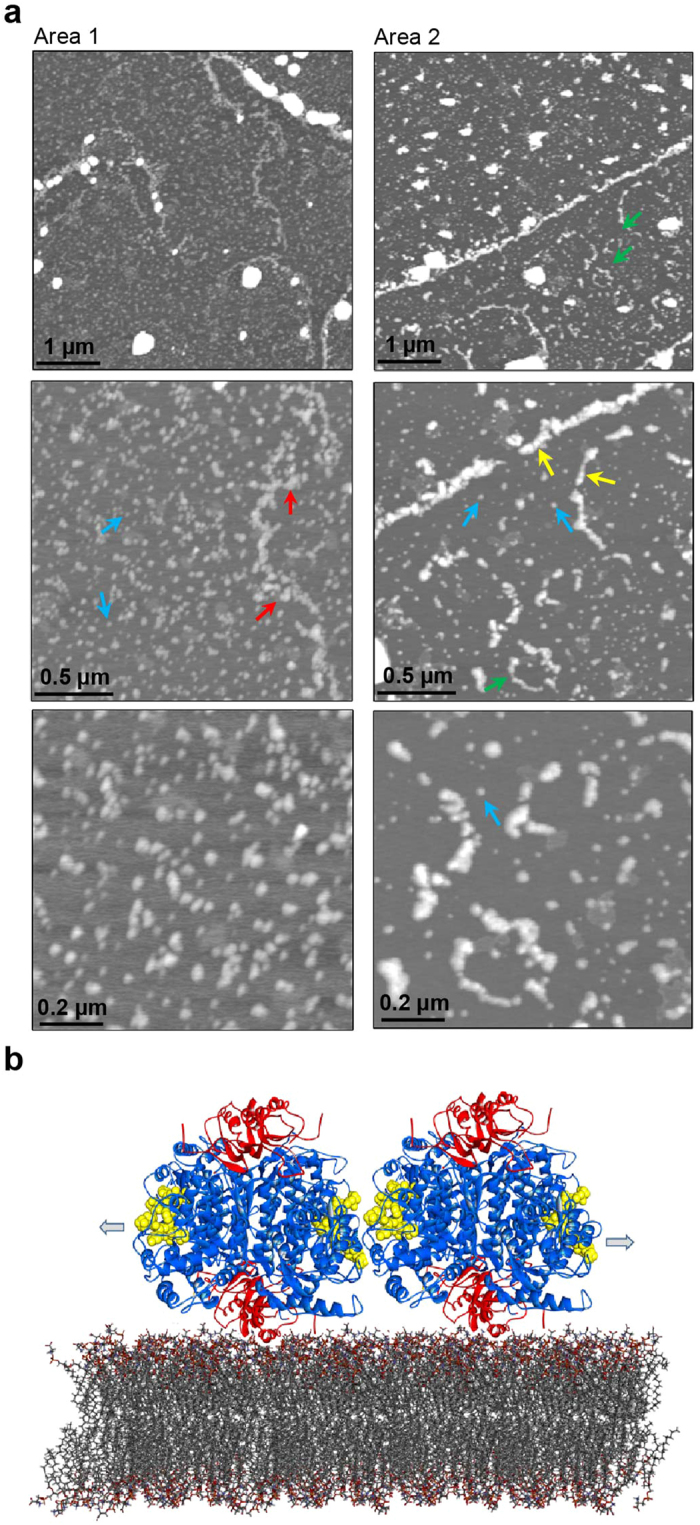
Amyloid aggregation of hTH1. (**a)** AFM height images of hTH1 deposited on PBPS:PC monolayer on mica substrate (3 nM subunit hTH1). hTH1 at pH 7.0 was injected under the compressed monolayer. Images of 5 μm, 2 μm and 1 μm scan sizes were acquired after 24 h. Arrows indicate different structures and aggregated species of hTH1 that were observed, such as large oligomers of hTH with ~40 nm in diameter (blue arrows), prefibrillar chains (yellow arrows), ring-like species with diameter >200 nm (green arrows), and clusters of these prefibrillar structures (red arrows). (**b)** Schematic representation of binding and aggregation of hTH1 in the membrane. Structural model of tetrameric TH^16^ with the regulatory domains (PDB id 2MDA) shown in red and the catalytic domains (PDB id 2XSN) in blue. The residues in yellow, CPK style, correspond to the surface exposed motif SVYFVS located in surface exposed β-strands. According to TANGO calculations^39^ this motif is characterized by high propensity to β-aggregation. The arrows indicate that these motifs likely can be engaged in intermolecular interactions between tetramers upon membrane-accumulation of TH.

Further insights into the conformational change, oligomerization and aggregation of hTH1 induced by membrane binding were obtained by AFM. Applying the Langmuir-Blodgett technique, hTH1 was injected under the pre-compressed PBPS:PC monolayer, thus when pulling up the hydrophilic mica, lipid head groups were oriented towards mica surface, leaving the interacting protein trapped between mica and lipid monolayer. This protocol has been found to be suitable to reflect physiological conditions, and ensure that the AFM measurements represent the protein-monolayer interactions^37^. We have also proven the suitability of this protocol in earlier AFM studies on phospholipid monolayers^38^. Consequently, we imaged an imprint of hTH1 on the lipid monolayer. Several areas of these samples were scanned at different scan sizes. As seen in Fig. 4a, height images show monolayer distortions of different topographies, with predominant round structures of ∼40 nm diameter in the *xy*-plane (blue arrows), corresponding to large oligomers of hTH1. Prefibrillar chains (yellow arrows), ring-shaped species of diameter >200 nm, reminiscent of annular protofibrils (green arrows) and clusters without specific shape (red arrows) were also observed.

Altogether, our results on the conformational determinants and changes involved in hTH1-membrane interaction support the membrane-enhanced aggregation of enzyme and concomitant disruption of membrane integrity. The initial membrane binding involves the N-terminal tails with propensity to form α-helices^20^,^21^ without prompting large conformational changes or embedment of hTH1 into the membrane, which nevertheless was sufficient to debilitate the integrity of synthetic bilayers (Fig. 1). Furthermore, time-course CD-monitoring and ThT fluorescence support that this initial phase also includes the slow cross-β type self-aggregation of hTH1, apparently seeding for a more ostensible amyloid aggregation. We applied the program TANGO^39^ to predict motifs with high propensity for this type of aggregation in the hTH1 sequence, finding that the motives of 81–86 (VLNLLF) in the regulatory domain, and those of 299–305 (FLASLAF) and 429–434 (SVYFVS) in the catalytic domain present a very high propensity for intermolecular cross-β interactions. Only the latter region in the catalytic domains is solvent exposed and forms β-strand with suitable orientation to be engaged in intermolecular interactions as indicated in the model presented in Fig. 4b.

### Effect of hTH1 on Mitochondrial Function and Cell Viability

**Figure 5:**
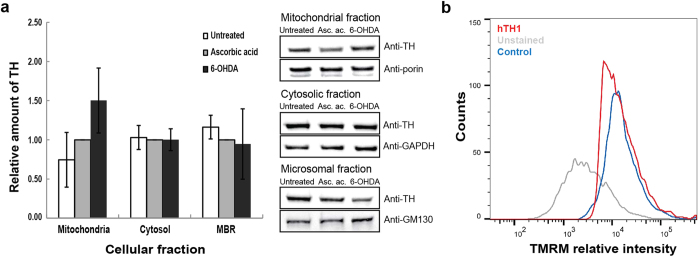
Interaction of TH with organelle membranes and its effect on mitochondrial membrane potential. (**a)** Relative amount of TH in PC12 cell fractions (left) and representative Western blots (right). PC12 cells were treated for 30 min with 75 μM of 6-OHDA prepared in ascorbic acid (Asc. ac.) solution. The graph shows the mean ± SD (n = 3) and representative blots of TH in mitochondrial, cytosolic and microsomal fractions in untreated, treated (6-OHDA in ascorbic acid), and relative to the reference (Asc. ac.; = 1) cellular samples. The mitochondrial fraction showed a fold-change of 1.54 ± 0.39 (p-value = 0.07). (**b)** PC12 cell mitochondrial membrane potential analyzed by using TMRM loading and flow cytometry after incubation with 10 μM hTH1 (red). Untreated, control mitochondria were also analysed (blue). The grey curve represents a profile of unstained mitochondria.

In order to probe if hTH1 inducing liposome-disruption could have some effect on dysregulated cellular systems, we used a procedure by Hanrott *et al*. to mimic early neuropathological events^40^. PC12 cells were treated with 75 μM 6-OHDA for 30 min, followed by an 18 h incubation in fresh media in order to prevent overly cell death. Under these conditions, the mitochondrial function of the cells was slightly compromised, but apoptosis had not yet been triggered, as indicated by staining with tetramethylrhodamine percolate (TMRM) and annexin-V markers in combination with flow cytometry (data not shown)^40^. Immunodetection of TH in subcellular fractionations showed an increase of TH in the mitochondrial fraction of treated cell cultures compared to control (Fig. 5a). It has been shown that α-syn aggregates at the mitochondrial membrane causing its disruption^41^. As TH was found to localize at the mitochondria surface^42^, we analyzed its effect on mitochondria functionality. As shown in Fig. 5b, TMRM loading into isolated mitochondria previously incubated with hTH1 is decreased compared to intact mitochondria.

**Figure 6:**
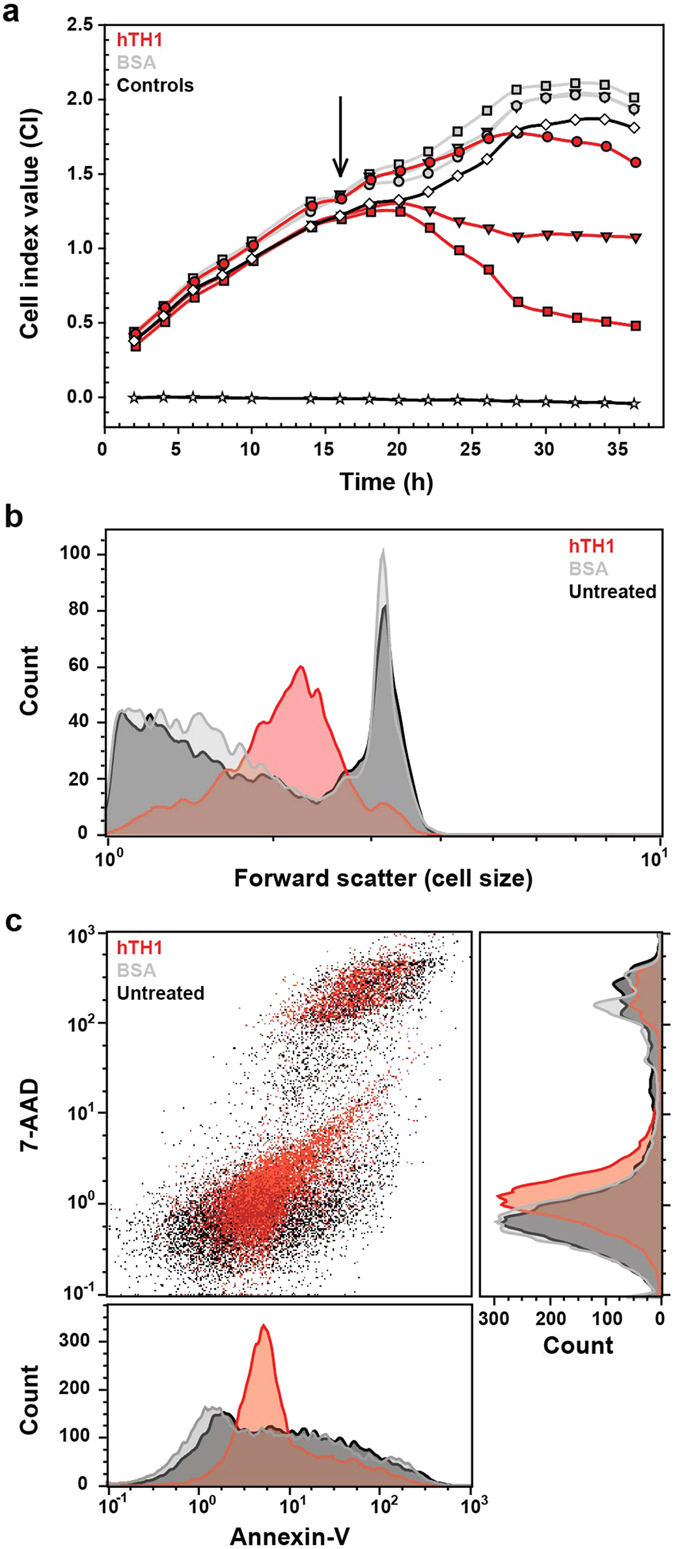
Extracellular hTH1 and cell viability. (**a)** Viability of HEK293 cell cultures monitored by impedance change upon addition of either hTH1 (red lines) or BSA-control (grey lines) at 200 nM (○), 2 μM (▽) and 5 μM (□) subunit concentrations. Cell (♢) and media (☆) controls (black lines) correspond to wells contai ning only HEK293 cells or culture media, respectively. The arrow indicates the addition of hTH1 or BSA. (**b)** Cell size measurement by forward scatter reveals a decrease in cell size when treated for 20 h with 5 μM hTH1 (red) in comparison to untreated (black) and 5 μM BSA-treated cells (grey). The cell size decreases and population becomes less defined in size when treated with hTH1, showing a broader size distribution than controls. (**c)** Top left chart represents the scatter plot of annexin-V *vs.* 7-AAD staining of hTH1 treated cells. Top right and bottom figures are projections of the scatter plot shown as histograms for 7-AAD and annexin-V, respectively. Annexin-V binding is higher in cells treated for 20 h with 5 μM of hTH1 (red) than in untreated (black) or 5 μM BSA-treated cells (grey). 7-AAD signal is also slightly increased on hTH1-treated cells in comparison to controls. The treatment with hTH1 results in a characteristic scatter plot distribution of the cell population assessed by annexin-V^high^ and 7-AAD^high^. Three independent experiments were performed, and a representative one is shown (**b,c**).

Given that necroptosis, a recently described controlled necrosis cell death pathway, is gaining importance in neurodegenerative diseases^43^,^44^, we wanted to investigate if a potential release and accumulation of TH in the extracellular environment due to cellular necrosis could further influence cellular viability and contribute to the spreading and amplification of toxicity. To this end, we monitored cell growth using real-time impedance after the addition of hTH1 to the media with HEK293 cells, which are commonly used cellular systems. For some proteins, it has been postulated that the extracellular concentration in necrotic situations can reach several-fold of normal physiological concentrations^45^. Thus, we used a wide (200 nM–5 μM) subunit concentration range to screen for any possible toxic effect of hTH1 in an *in vitro* system. Figure 6a shows a hTH1 concentration dependent decrease of cell viability, whereas similar concentrations of BSA, a plasma protein of 66 kDa subunit, which is comparable with 56 kDa for hTH1, did not show this effect (Fig. 6a). In order to distinguish if the reduction of cell viability was due to necrosis or apoptosis we stained our samples with 7-aminoactinomycin D (7-AAD) and annexin-V and analyzed them using a Muse flow cytometer. The forward scattering showed a cell size decrease after incubation with hTH1 compared to untreated cells or BSA-treated samples (Fig. 6b). This may reflect cell shrinkage due to cell death, consistent with the real-time impedance results (Fig. 6a). Comparing 7-AAD uptake and annexin-V binding for the different conditions, the signal from annexin-V is higher in the hTH1 treated cells than in untreated cells or the BSA-treated samples (Fig. 6c), however, 7-AAD signal increases only slightly (Fig. 6c). Interestingly, the scatter plot of 7-AAD *vs*. annexin-V for the population of hTH1 treated cells shows a characteristic shape pointing in the direction of high levels of annexin-V and 7-AAD signals (Fig. 6c). Altogether, our data indicate that apoptosis processes could be triggered after cells are exposed to hTH1, which is compatible with necroptosis due to the crosstalk of the two pathways^46^.

## Discussion

A large number of surface active proteins spontaneously accumulate or adsorb on a membrane through structural or electrostatic changes^3^. Some of these proteins, when accumulated, can cause membrane destabilization and increased permeability, which can be associated to pathological processes^11^,^47^.

In previous studies on membrane binding of TH, it has been hypothesized that this interaction might be a mechanism coordinating the synthesis of neurotransmitters at the site of their accumulation at the synaptic vesicles^19^. However, we have shown that the interaction of hTH1 with negatively charged membranes in fact results in a decreased specific activity of this enzyme^20^,^21^. Furthermore, the enzyme is characterized as largely soluble and cytoplasmic, while physiological association with membranes appears to occur through the binding to other partners that certainly anchor to the membrane^18^,^20^. Thus, there is a sound possibility that the direct interaction of TH with membranes might not be a physiologically relevant mechanism, but rather a dysregulated interaction with potential pathogenic consequences. We therefore focused in this work not only on the conformational effects that the protein undergoes upon direct binding to the membrane, but also on the outcome concerning membrane morphology and permeability as well as cellular viability. Biological membranes are highly complex but unilamellar phospholipid vesicles constitute appropriate *in vitro* models to investigate molecular mechanisms involved in protein-membrane interactions^20^,^48^. Unilamellar membrane models (liposomes) of varying size and phospholipid composition, modulating the charge and fluidity of the membrane, aim to reflect the properties of relevant cellular organelles and vesicles^49^. Small unilamellar vesicles (SUVs) made of DOPE:DOPS:DOPC (5:3:2) have been used to suitably mimic the size, membrane and fluidity of synaptic vesicular membranes in studies of the conformations of vesicle-bound α-syn^31^,^32^. In comparative tests with LUVs of PBPS:PC and DOPE:DOPS:DOPC we observed that TH binds to both compositions, but only induces leakage to the PBPS:PC bilayer, which represents neuronal membranes in general. The loss of the membrane integrity and increased permeability upon hTH1 membrane-binding was also confirmed by confocal microscopy on GUVs of PBPS:PC. This phospholipid mixture is more heterogeneous than DOPE:DOPS:DOPC, which only includes 18:1 di-oleic fatty acids and, interestingly, PBPS:PC also contains polyunsaturated fatty acid hexaenoic acid (DHA; 22:6) in the PBPS phospholipid fraction (11%). Disruptions in the bilayer caused by specific lipid domain formation due to heterogeneous phospholipid head and fatty acid composition may act as seeding points that favour leakage and protein oligomerization^38^. In addition, in the case of α-syn, it has been shown that the presence of DHA has an impact on the binding and oligomerization of the protein at the membrane^50^ and concomitant increase of the membrane permeability^51^. Thus, increased heterogeneity and the presence of DHA might be important to explain the higher disruption propensity of LUVs made of PBPS:PC.

The N-terminal extension to the regulatory domain (residues 1–43 of hTH1; TH-(1–43)) includes the main determinants for the interaction with membranes (ref. 21 and this work). Experimental^20^ and computational^28^ studies on TH-(1-43) peptide revealed an increased α-helical content in this stretch when bound to negatively charged membranes, a change that may be less easy to observe in the full-length enzyme, a tetramer of 497 amino acids per subunit. Many other protein-membrane interactions appear to involve amphipathic helices^3^, and altogether, our results support a model in which hTH1 associates to the membrane via the α-helical N-terminus and aggregates through inter-tetramer cross-β interactions involving the catalytic domains (Fig. 4b). As outlined in our model (Fig. 4b), hTH1 may bind to the membrane in its tetrameric form and – over time – aggregate into prefibrillar chains or rings through β-aggregation of the surface exposed SVYFVS motives, facing the same motives in neighbouring tetramers when oriented in the membrane. The size and shape of the resulting structures (Fig. 4a) resemble protofibrillar aggregates formed by other proteins involved in neurodegenerative diseases, such as α-syn and amyloid peptides, among others^52^,^53^. It has been shown that negatively charged lipid membranes drive the accumulation of amphipathic proteins, causing a molecular crowding at their surface and leading to protein unfolding and amyloid aggregation^54^.

Protein aggregation is a relatively complex process that might require several steps until insoluble aggregates are formed. One of the most characteristic highly ordered conformation is amyloid fibrils, formed out of protofibrils. It has been shown that there is a different toxicity among aggregate species. Immature protein aggregates, especially protofibrils, seem to show the highest toxicity, whereas fibrils appear to be considerably less toxic^55^. In the case of α-syn, prefibrillar and oligomeric conformations have extensively been shown to have a much higher membrane-disruptive pore-forming activity than monomeric conformers^56^. We did not observe pores or annular oligomers by AFM, and membrane thinning upon accumulation of protein at the surface might be the mechanism behind the permeabilizing effect of hTH1 on the membrane, at least at initial stages^57^,^58^. Indeed, TH has been shown to localize at the mitochondrial surface and the relatively short exposure of PC12 cells to neurotoxin already caused an increase of TH content in the mitochondrial fraction. This re-localization of TH appears to affect the mitochondrial potential, as shown in experiments with isolated mitochondria, which could contribute to PD pathology as mitochondrial dysfunction is the key to the disease and appears to be an early stage event^59^. Moreover, it has recently been suggested that different types of cell death, such as apoptosis, autophagy, and necroptosis, the programmed necrosis, can co-exist and crosstalk in PD^44^,^46^. Programmed necrosis of dopaminergic neurons could involve the release of TH, which would then come in contact with plasmatic membrane of surrounding cells. Our cellular viability assays suggest that hTH1 does not have an immediate lethal effect on cells, however it compromises their viability over time in a concentration-dependent manner, apoptotic and/or necroptotic processes. Even more, the breach of the membrane integrity might even result in TH leakage and a TH release into the extracellular environment, increasing TH levels in a self-amplifying positive feedback, potentially though a necroptotic pathway, which may trigger a spiraling process of cell death. As for α-syn and BAX (BCL2-associated X protein)^60^,^61^, cellular toxicity could be associated with the harmful effect of TH on membrane integrity.

Up to date, TH has not been identified itself as a major contributor to the neurodegenerative process despite its large abundance in dopaminergic cells. This is most probably related to the fact that TH has not appeared as a candidate gene for PD in genome-wide association studies and has not been reported to accumulate in Lewy bodies of neurodegenerating cells. Nevertheless, some earlier reports have suggested a direct pathogenic role of TH in PD through different mechanisms^25^,^26^,^27^. Moreover, several recent reports have pointed to gain of function upon TH amyloid aggregation in cellular systems, notably when the ubiquitin-dependent proteasome system is dysregulated^62^. Nevertheless, an involvement of amyloid aggregation at the membrane and its effect on cellular toxicity via disruption of membrane integrity has not earlier been shown. Due to high abundance of TH in the brain tissues and its propensity to self-assemble into amyloid structures on intra- and external cellular membranes this pathogenic effect may be considered as a contributing factor to progression of the neurodegenerative cascade in PD. Further studies will be necessary to properly characterize the correlations between the *in vitro* and *in vivo* effects elicited through TH interaction with membranes, and better understand the associated pathogenic mechanisms and role in PD.

## Methods

### Materials

Avanti Polar Lipids (Alabaster, USA) supplied porcine brain phosphatidylserine (PBPS), egg yolk phosphatidylcholine (PC), dioleoyl phosphoethanolamine (DOPE), dioleoyl phospho-L-serine (DOPS) and dioleoyl phosphocholine (DOPC). All other reagents were purchased from Sigma-Aldrich (St. Louis, MO, USA) unless stated otherwise.

### Protein Expression and Purification

Full-length human TH type 1 (hTH1; sp P07101-3 in UniProtKB/Swiss-Prot) was expressed in BL21-CodonPlus Competent Cells (Agilent) as a His-tagged ZZ-fusion protein ((His)_6_-ZZ-TH)) as described elsewhere^33^. The tag was cut with His-tagged TEV and pure hTH1 (in 20 mM Na-Hepes, 200 mM NaCl, pH 7.0) was obtained after TALON column purification (see Supplementary).

### Limited Proteolysis by Trypsin

Tryptic digestion of hTH1 (6 mg/mL) was performed using a trypsin:protein ratio of 1:200 (w:w) at 25 °C for 1 min in 20 mM Na-Hepes, 200 mM NaCl, pH 7.0 and was stopped with 1.5 μg/mL trypsin soybean inhibitor. N-terminal peptides were eliminated using a Superdex 200 10/300 column (GE Healthcare).

### Phospholipid Large Unilamellar Vesicle Preparation

In order to prepare liposomes, as LUVs, chloroform-dissolved PBPS and PC phospholipids were mixed at 1:1 ratio to prepare the PBPS:PC mixtures and DOPE, DOPS and DOPC at 5:3:2 ratio for the DOPE:DOPS:DOPC mixtures, and chloroform was removed by lyophilization. Lipids were resuspended in buffer (as specified in each section) and incubated at 25 °C, 120 rpm for at least 2 h. Leakage measurement buffers also contained 12.5 mM ANTS fluorochrome and 45 mM DPX quencher. The hydrated phospholipid solutions were frozen and thawed seven times followed by extrusion with an Avanti Mini-Extruder through polycarbonate membranes with a 100 nm diameter pore-size. Unencapsulated ANTS and DPX were removed with a Sephadex G-75 column. GUVs were prepared using the same lipid mixture used for LUVs, but glucose (1:20 ratio) was added before forming the lipid film and vacuum-drying it for 2 h. The swelling/hydration was performed in 10 mM Na-Hepes, 150 mM NaCl pH 6.0, at room temperature overnight. Phospholipid concentration in the vesicle preparations was determined according to Fiske and Subbarow^63^.

### Leakage Assay

ANTS fluorescence emission (510 nm) was recorded continuously for 1 h with 1-min intervals at 37 °C on a 96-well plate and a Synergy H1 Hybrid Reader (BioTek) after 355 nm excitation. Experiments were performed in triplicates using either PBPS:PC or DOPE:DOPS:DOPC LUVs, with final concentrations of 0.8 mM in phospholipid and up to 30 μM subunit of hTH1 or 10 μM hTH1_trunc_ in 10 mM Na-Hepes, 150 mM NaCl, pH 7.0. Samples lacking hTH1 served as control. 2 mM Triton X-100 was added at the end of each time scan. Parallels were averaged after processing the percentages of the release and subtracted from the controls (see Supplementary).

### Confocal Microscopy

Imaging was performed at 37 °C on a Leica TCS SP5 confocal microscope using a HCX PL APO CS 100.0x/1.40 N/A oil-immersion objective with argon and HeNe laser lines (488 nm and 561 nm, respectively). 1 μM rhodamine B labelled-GUVs were embed in 10 mM Na-Hepes, 150 mM NaCl, pH 6.0 containing 3 μM calcein, 0.5% agarose and after focusing on single GUVs, 20 μM hTH1 was carefully added. 512 × 512 pixel images were taken for 36 min with 2-min intervals. Addition of 10 mM Na-Hepes, 150 mM NaCl, pH 6.0 served as the control.

### Circular Dichroism

CD measurements were performed with a Jasco’s J-810 spectropolarimeter at 37 °C in the far-UV spectral range using a 0.1 nm data pitch, 2 nm band width, and a 20 nm/min scan speed. Samples were analyzed in 1 mm quartz cell and 2 accumulations of the 195–260 nm spectra were recorded for hTH1 (4 μM subunit) in the presence or absence of PBPS:PC liposomes (0.32 mM phospholipid concentration) in 10 mM Na-Hepes, 150 mM KF, pH 7.0. Time-resolved experiments are done under identical conditions. Base-line spectra of buffer or liposomes lacking protein were subtracted from the sample spectra, converted into mean ellipticity, averaged and further analyzed with the CDNN algorithm^34^ (see Supplementary).

### Dynamic Light Scattering

DLS was performed on a Malvern Zetasizer Nano ZS with a HeNe laser at 633 nm and a fixed scattering angle of 173° (back scatter). Scans of hTH1, PBPS:PC liposomes, or both together were taken in 10 mM Na-Hepes, 150 mM NaCl, pH 7.0, and incubated for 30 min at 37 °C. The final concentrations for hTH1 and PBPS:PC liposomes were 4 μM subunit and 0.32 mM phospholipid, respectively. Intensity and volume size distribution curves were analyzed using Malvern DTS software.

### Intrinsic Tryptophan Fluorescence

hTH1 (2 μM subunit) intrinsic tryptophan fluorescence spectra in the presence or absence of PBPS:PC LUVs (0.16 mM in phospholipid) were acquired on a Cary Eclipse spectrophotometer in 10 mM Na-Hepes, 150 mM NaCl, pH 7.0 at 37 °C with an excitation wavelength of 295 nm. Emission spectra were recorded at 310–400 nm at a scan rate of 600 nm/min, with 10- and 20-nm slit widths for excitation and emission pathways, respectively. Base-line spectra of buffer or liposomes lacking protein were subtracted from the sample spectra. A total of three independent measurements each performed in triplicates were averaged. λ_em max_ values were obtained from four-parameter log-normal function fits to the data as described in Burstein *et al*.^64^. hTH1 insertion into the membrane was studied by acrylamide Trp quenching, using a 0–200 mM acrylamide concentration range to 2 μM subunit hTH1 in the presence or absence of PBPS:PC liposomes (1:80 protein:lipid molar ratio). The values obtained were corrected for solvent blank, dilution, and sample absorbance. Quenching rate constants were determined from the Stern-Volmer equation: F_0_/F = 1 + *K*_sv_[Q], where F_0_ and F are the fluorescence intensities in the absence and presence of the quencher (Q), respectively. *K*_sv_ is the Stern-Volmer quenching constant, which is a measure of the accessibility of Trp to acrylamide.

### Atomic Force Microscopy

Lipid monolayers were deposited using Langmuir-Blodgett transfer according to Baumann *et al*.^38^. Briefly, freshly cleaved mica was immersed in the KSV Minitrough filled with 10 mM Na-Hepes, 150 mM NaCl, pH 7.0 at 37 °C before 20 μL of 1 mg/mL PBPS:PC (1:1) dissolved in chloroform was spread at the air-water interphase. The lipid monolayer was compressed at 5 mm/min to a surface pressure of 30 mN/m, which was recorded by a Wilhelmy plate. After 20 min stabilization, 3 nM hTH1 was injected under the compressed monolayer, allowed 30 min of equilibration, and then the mica was vertically pulled (5 mm/min) from the subphase buffer through the lipid film into air. Imaging was performed using a MFP-3D-Bio^TM^ atomic force microscope (Asylum research) in AC mode in air at room temperature using silicon cantilevers (AC240TS, Olympus) with a resonant frequency of ∼55 KHz and a ∼2 N/m spring constant. 256 × 256 pixels images were captured at a 0.5–1 Hz line scan rate.

### ThT Fluorescence

ThT fluorescence emission was recorded at 482 nm in a 96-well plate for 24 h at 37 °C on a Synergy H1 Hybrid Reader (BioTek) after excitation at 440 nm. Intensity data of hTH1 and hTH1_trunc_ (10 μM subunit) with 20 μM ThT in the presence or absence of PBPS:PC liposomes (0.8 mM in phospholipid) were acquired in 10 mM Na-Hepes, 150 mM NaCl, pH 7.0. Samples lacking protein were used as controls and subtracted. Three parallel measurements were performed.

### Cell Culture and 6-OHDA Treatment

HEK293 cells were grown in DMEM supplemented with 5% fetal bovine serum, 2 mM glutamine, 100 U/mL penicillin and 100 μg/mL streptomycin in a 5% CO_2_ and 37 °C atmosphere. Rat pheochromocytome adherent PC12 cells (ATCC-CRL-1721.1; <8 passes) were grown in RPMI-1640 medium with 10% horse serum (Gibco) and 5% fetal bovine serum supplemented with 2 mM glutamine, 100 U/mL penicillin and 100 μg/mL streptomycin. 24 h after seeding, PC12 cells were exposed for 30 min to 75 μM 6-OHDA (in 5.7 mM ascorbic acid) and grown 18 h in fresh media.

### Subcellular Fractionation and Western blot

Subcellular fractionation of drug-treated cultures were performed essentially according to ref. 65. Fractions were analysed by 10% TGX SDS-PAGE and proteins blotted onto nitrocellulose membranes using TransBlot turbo (Bio-Rad). Blots were incubated with anti-TH (rabbit; Thermo Scientific), anti-GM130 (mouse; BD Transduction Laboratories), anti-GAPDH (rabbit, Abcam) or anti-porin (mouse; Abcam) primary antibodies. Horse radish peroxidase coupled to anti-rabbit or anti-mouse antibodies, ChemiDoc and Image Lab software (Bio-Rad) were used for chemiluminiscence visualization and analysis of blots. The intensity of target proteins was standardized with the corresponding loading control for each subcellular fraction. Treated samples were compared to untreated, which were normalized to a value of 1. The sample size in all cases was *n* = 3. Results are given as mean value ± SD and a two-way comparison using the *t-*test was performed in order to determine statistical significance.

### Real-time Cell Viability Monitoring by Electric Cell-Substrate Impedance Sensor System

A xCELLigence Real-Time Cell Analyzer Single Plate (RTCA SP) system with E-16 plates (Roche Applied Science) was used to monitor cell growth by electrical impedance, which is displayed in arbitrary cell index *units (CI).* After culture media was equilibrated to room temperature, background reading was performed at 37 °C, in 5% CO_2_ humidified incubator according to manufacturer’s instructions. 20,000 cells/well were seeded, allowed to settle for 30 min, and then monitored for 16 h pre-exposure. Then hTH1 or BSA (200 nM, 2 μM and 5 μM) were diluted into culture media and impedance recordings were continued. Control wells contained only culture media. Experiments were performed in duplicates.

### Flow Cytometry Analysis

PC12 cells incubated with 50 nM of TMRM and Annexin V–Alexa 488 (Invitrogen) were collected by centrifugation and resuspended in PBS for analysis on an AccuriC6 flow cytometer (BD Biosciences; FL-1 and FL-3 setting, 10,000 events/analysis). Alternatively, isolated mitochondria prepared as described in ref. 65 and resuspended in 250 mM sucrose in PBS with protease inhibitor (Roche) were incubated with 10 μM hTH1 for 20 min and then loaded with TMRM for 20 min, prior to analysis.

Muse Annexin-V and Dead Cell Assay (Millipore) was used to analyze apoptosis caused by addition of hTH1 to the cell culture. 150,000 HEK293 cells were grown overnight before 20 h incubation with 5 μM of hTH1 or BSA (control) in media. Dead cells were collected from the culture media by centrifugation and resuspended together with the remaining cells collected by trypsinization. The final cell suspension was incubated for 30 min with Muse Annexin-V and Dead Cell reagent. Samples were analyzed using the Muse Cell Analyzer flow cytometer collecting 10,000 events per sample, recording forward scatter and the fluorescence signals from the annexin-V-conjugate and 7-AAD. All flow cytometry data was processed using FlowJo V10. Three independent experiments were performed, and a representative one is shown.

## Additional Information

**How to cite this article**: Baumann, A. *et al*. Tyrosine Hydroxylase Binding to Phospholipid Membranes Prompts Its Amyloid Aggregation and Compromises Bilayer Integrity. *Sci. Rep.* **6**, 39488; doi: 10.1038/srep39488 (2016).

**Publisher's note:** Springer Nature remains neutral with regard to jurisdictional claims in published maps and institutional affiliations.

## Supplementary Material

Supplementary Information
