# Supplementary material for: Tyrosine Hydroxylase Binding to Phospholipid Membranes Prompts Its Amyloid Aggregation and Compromises Bilayer Integrity
Source: Sci Rep. 2016 Dec 22;6:39488. doi: 10.1038/srep39488 (PMC5177901; doi:10.1038/srep39488)
Supplement: Supplementary Information [file srep39488-s1.pdf]

# <u>Supplementary</u>

# Tyrosine Hydroxylase Binding to Phospholipid Membranes Prompts Its Amyloid Aggregation and Compromises Bilayer Integrity

**Anne Baumann[1,2,*], Ana Jorge-Finnigan[1,3], Kunwar Jung-KC[1], Alexander Sauter[1,4], Istvan Horvath[5], Ludmilla A. Morozova-Roche[5] and Aurora Martinez[1,3,*]**

[1]Department of Biomedicine, University of Bergen, 5009 Bergen, Norway

[2]Division of Psychiatry, Haukeland University Hospital, 5021 Bergen, Norway

[3]K.G. Jebsen Centre for Neuropsychiatric Disorders, University of Bergen, 5009 Bergen, Norway

[4]Department of Clinical Dentistry, University of Bergen, 5009 Bergen, Norway

[5]Department of Medical Biochemistry and Biophysics, Umeå University, 90187 Umeå, Sweden

*To whom correspondence should be addressed: Anne.Baumann@uib.no or Aurora.Martinez@uib.no

## Methods

*Protein Expression and Purification*

Full length human TH type 1 (hTH1; sp P07101-3 in UniProtKB/Swiss-Prot) was expressed in BL21-CodonPlus Competent Cells (Agilent) as a his-tagged ZZ-fusion protein ($(His)_6$-ZZ-TH)) from the pET-ZZ-1a vector[1,2]. Protein was expressed at 28°C in autoinduction medium containing 100 µg/mL kanamycin. Bacteria were harvested by centrifugation (4000 rpm, 20 min, 4°C) and the pellets were kept at -80°C until further use. Purification of hTH1 from bacterial pellets was performed as follows: Pellets were resuspended in 50 mM Na-phosphate, 300 mM NaCl, pH 7.0 for purification on TALON® metal affinity columns (Clontech) containing 1 mM PMSF. The cells were disrupted by sonication (Vibra-Cell™, Sonics & Materials, Inc.) and clarified extract was applied to the respective resin. The $(His)_6$-ZZ-hTH1 fusion protein was eluted using buffer supplemented with 150 mM imidazole and concentrated with 50 kDa cut-off Amicon® Ultra Centrifugal filters (Millipore Corporation). Imidazole was removed using PD-10 columns (GE Healthcare) before the fusion protein

was cut using His-tagged TEV. To remove the ZZ-fusion partner and TEV from hTH1, the sample was applied to a second TALON® column, leaving pure hTH1 in 20 mM Na-Hepes, 200 mM NaCl, pH 7.0. hTH1 concentration was measured using an absorption coefficient of $A_{280\ nm}$ (1 %) = 7.32 L/g $cm^{-1}$, and is provided as the subunit concentration.

*Leakage Assay*

To estimate the end-point for full disruption of the liposomes, the non-ionic detergent Triton X-100 was used at the end of each time scan. A final concentration of 2 mM of this detergent in the cuvette ensured lysis of the liposomes and release of encapsulated contents. Leakage was thus evaluated as a percentage of complete release by Triton X-100. The initial intensity of the liposome solution was set to 0 % leakage and the final intensity after adding Triton X-100 corresponded to 100 % leakage. Parallels were averaged after processing the percentages of the release and subtracted from the controls. Fitting to a sigmoidal, logistic 3 or 4 parameter function ($y = a / (1 + (x/x_0)^b)$ or $y = y_0\ a / (1 + (x/x_0)^b)$), where $y_0$ is the offset from zero, *a* the amplitude of the curve, x the time, $x_0$ the time of the sigmoid midpoint and b the rate constant), was applied in order to compare the leakage (%) between the different hTH1 concentrations.

*Circular Dichroism*

The mean ellipticity was determined using the formula $[\theta] = \theta/(10 \cdot c \cdot l)$, where θ is the ellipticity (mdeg), 10 is a scaling factor, c is the protein concentration (M) and l is the path length of the cuvette (cm). Three parallels were averaged after subtraction of baseline spectra and calculation of mean ellipticity. Estimation of the secondary structure was performed by using the program CDNN[3].


1 Kleppe, R. et al. Phosphorylation dependence and stoichiometry of the complex formed by tyrosine hydroxylase and 14-3-3gamma. *Mol. Cell. Proteomics* **13**, 2017-2030 (2014).

2 Bezem, M. T. et al. Stable preparations of tyrosine hydroxylase provide the solution structure of the full-length enzyme. *Sci. Rep.* **6**, 30390 (2016).

3 Böhm, G., Muhr, R. & Jaenicke, R. Quantitative analysis of protein far UV circular dichroism spectra by neural networks. *Protein Eng.* **5**, 191-195 (1992).
